# A Retrospective Study Comparison Between Stenting and Standardized Medical Treatment for Intracranial Vertebrobasilar Stenosis in a Real-World Chinese Cohort

**DOI:** 10.3389/fneur.2021.629644

**Published:** 2021-05-31

**Authors:** Guanzeng Li, Peng Yan, Yuanyuan Zhao, Shan Li, Yuan Xue, Yuanyuan Xiang, Xiaohui Liu, Jifeng Li, Qinjian Sun

**Affiliations:** ^1^Department of Neurology, Shandong Provincial Hospital, Cheeloo College of Medicine, Shandong University, Jinan, China; ^2^Department of Neurology, Liaocheng People's Hospital, Liaocheng, China; ^3^Department of Neurology, Shandong Provincial Hospital Affiliated to Shandong First Medical University, Jinan, China

**Keywords:** real-world, standardized medical treatment, intracranial artery stenosis, stenting angioplasty, vertebrobasilar artery

## Abstract

**Background:** To date, there has been no consensus regarding the benefits of percutaneous transluminal angioplasty and stenting (PTAS) vs. those of standardized medical treatment (SMT) for patients with symptomatic intracranial vertebrobasilar stenosis (IVBS). The purpose of this retrospective study was to compare the effects of PTAS or SMT on symptomatic IVBS in a real-world Chinese population.

**Methods:** We included 238 patients with ischemic stroke caused by IVBS stenosis who were admitted to Shandong Provincial Hospital Affiliated to Shandong University between September 2012 and May 2018; 62 of these patients were treated with SMT and 176 underwent PTAS. Ischemic stroke in the territory of the responsible artery, hemorrhage, and death within 1 year were recorded as primary endpoints. Secondary endpoints included assessment of stroke severity and the incidence of re-stenosis. The primary endpoint rates were compared between the PTAS and SMT groups at 7 days, 1, 6 months, and 1 year.

**Results:** In the PTAS group, the success rate of stent placement was 98.9%. During the entire trial, except for 7 days, the SMT group had a higher frequency of primary endpoint events than did the PTAS group. The primary endpoint was 17.7% (11/62) vs. 8.6% (15/174) at 1 month (*p* = 0.049), 29% (18/62) vs. 14.4% (25/174) at 6 months (*p* = 0.01), and 32.2% (20/62) vs. 17.2% (30/174) at 1 year (*p* = 0.013). The restenosis rate of the target lesion was 13.8%; 60% were symptomatic restenosis and 40% were asymptomatic restenosis. The rate of severe stroke at 1 year after PTAS was 0%, while that in the SMT group was 9.7%.

**Conclusions:** In a real-world Chinese cohort, PTAS for patients might be superior to SMT, and provide better long-term neurological function recovery and lower disability rate.

## Introduction

Intracranial atherosclerotic stenosis (ICAS) is an important cause of ischemic stroke worldwide. In Caucasians, 5–10% of strokes are caused by ICAS, while this ratio is 30–50% in Asian population (1). Approximately 20% of ischemic strokes are caused by vertebrobasilar artery stenosis (2). Compared with anterior circulation stenosis, intracranial vertebrobasilar artery stenosis (IVBS) leads to a higher risk of occurrence and recurrence of transient ischemic attack (TIA) or cerebral infarction (3–5). A study showed that patients with symptomatic IVBS had poor prognosis, high mortality, and high disability rate (4). It has been reported as high as 33% recurrence in the first month with the best medical therapy (5). Other studies, including patients with severe IVBS, have shown that despite aggressive medical treatment, the annual incidence of stroke remained between 10 and 28.7% (6–8).

Percutaneous transluminal angioplasty and stenting (PTAS) is another method of treating ICAS. The Stenting and Aggressive Medical Management for Preventing Recurrent Stroke in Intracranial Stenosis (SAMMPRIS) and the Vitesse Intracranial Stent Study for Ischemic Stroke Therapy (VISSIT) studies are the two prominent randomized clinical trials of intracranial stenting (9, 10). These studies showed no advantage of stenting over medical treatment for patients with ICAS, partly due to the high complication rate. However, a recent post-marketing surveillance trial (WEAVE trial) showed a 2.6% periprocedural stroke, bleeding, and death rate in patients with symptomatic ICAS lesions of 70–99% who underwent stenting angioplasty with the Wingspan stent (11). In the WOVEN trial, the natural extension of the WEAVE trial, stroke, or death rate was 8.5% at 1-year follow-up (12). A meta-analysis of symptomatic IVBS found that the annual stroke or death rate in the endovascular treatment group was approximately 8.9% (3). Therefore, it appears that the outcomes of endovascular procedures for symptomatic IVBS have been inconsistent. In addition, several studies in Asia showed inconsistent results of stenting vs. medical treatment for IVBS (13, 14). In the real world, the effect of PTAS on symptomatic IVBS remains elusive. Therefore, the purpose of this retrospective study was to compare the effects of PTAS and standardized medical treatment (SMT) in patients with IVBS in a real-world setting at a single center in China.

## Materials and Methods

### Patient Selection

Detailed clinical information was obtained from the Department of Neurology at Shandong Provincial Hospital, Cheeloo College of Medicine, Shandong University between January 2012 and December 2018. All patients were from Shandong Province, China. This retrospective study was a single-center study. This study was reviewed and approved by the ethical standards committees on human experimentation at Shandong Provincial Hospital, Cheeloo College of Medicine, Shandong University.

The inclusion criteria were as follows: (1) age of 18–80 years; (2) digital subtraction angiography (DSA) revealing that the stenosis of the intracranial vertebrobasilar artery was > 70%. The responsible arteries were the intracranial segment of the vertebral artery or the basilar artery. The degree of stenosis was measured using the Warfarin-Aspirin Symptomatic Intracranial Disease (WASID) trial method (5); and (3) TIA attack or cerebral infarction was related to posterior circulation. The typical symptoms of posterior circulation TIA include dizziness, numbness of limbs or face, weakness of limbs, vomiting, diplopia, hemianopia, gait disorder, temporary loss of consciousness, and falls. Cerebral infarction refers to magnetic resonance diffusion limitation in the cerebellum, brainstem, occipital lobe, and other posterior circulation areas.

Exclusion criteria were as follows: (1) severe systemic diseases or unsuitable or intolerable dual antiplatelet therapy; (2) non-atherosclerotic stenosis such as artery dissection, arteritis, or moyamoya disease, confirmed by DSA and high-resolution magnetic resonance; (3) international standardized ratio (INR) > 1.5, and uncorrectable bleeding tendency; (4) failure to follow up in time and life expectancy <5 years.

SMT group was comprised of those who suffered first onset of posterior circulation TIA or cerebral infarction and those who had recurrent events without SMT but refused PTAS. The indication for PTAS was posterior circulation TIA or occurrence of cerebral infarction despite SMT, and despite the first attack of posterior circulation TIA or cerebral infarction, stenting was required.

### Treatments

#### Standardized Medical Treatment

The medications in the SMT and PTAS groups were identical: aspirin 100 mg and clopidogrel 75 mg were given daily orally for 90 days in the SMT group and 180 days in the PTAS group after the onset of treatment. Thereafter, the administration of one antiplatelet agent was stopped. Statins (atorvastatin 20 mg or rosuvastatin 10 mg) were administered orally daily after the onset of treatment. All patients were treated with standard management of vascular risk factors, including systolic blood pressure <140 mmHg (diabetic patients <130 mmHg), and statins to lower low-density lipoprotein (LDL) levels <1.80 mmol/L, and blood glucose <7 mmol/L. We advised all patients to quit smoking, engage in sufficient exercise, and other lifestyle changes.

#### PTAS Procedure

PTAS was performed by a neurointerventionist, Qinjian Sun, who had been engaged in neurointerventional therapy for 10 years and had completed more than 500 cases of PTAS for ICAS. He had experience in more than 50 cases of intracranial artery stents before this study. He was credentialed to participate in this study based on a review of 20 consecutive intracranial angioplasty and stenting cases, at least five of which must have used the Wingspan system in order to reduce the occurrence of technical complications. The degree of stenosis before and after intervention was determined according to the WASID (5) criteria in DSA. Antiplatelet aggregation drugs were started 3–5 days before PTAS, along with clopidogrel 75 mg, and aspirin 100 mg orally. It was recommended to fast on the day of PTAS, but not to stop the administration of the antiplatelet drug.

The interventional procedures were performed under general anesthesia. Using the modified Seldinger technique, a 6-French vascular sheath was placed in the right femoral artery. A bolus of 5,000 units of heparin was administered. The 6-French Mach 1 guiding catheter (Boston Scientific, US) was placed into the distal V2 segment of the vertebral artery to provide sufficient support. A guiding catheter was used to perform the responsible artery angiography. The degree of stenosis was calculated based on the adjacent distal normal blood vessel diameter (WASID standard). The guide catheter containing the contrast agent was used as a reference, and the blood vessel diameter was calibrated through the DSA workstation.

The choice of the type of stent (Gateway-Wingspan system, Stryker, US; or Apollo stent system, Microport, China) depended on the arterial pathway, lesion morphology, and operator discretion. Operator was instructed to choose the devices based on the need of a patient individually. This decision of tailored stenting would primarily take into consideration the ease of vascular access and lesion morphology. According to our experience, the Gateway-Wingspan system is more suitable for patients with tortuous vascular access because the system has better flexibility in traversing curvature. Compared with the Gateway-Wingspan system, the Apollo stent (a bare-metal balloon-expandable intracranial Stent) is more rigid; however, it is a good choice for patients with smoother access because the delivery of the balloon-expandable stent does not require replacement and requires less surgical time (Table 1). If the Gateway–Wingspan system was selected, the gateway balloon was used to pre-expand the stenosis before stent implantation. The recommended diameter of the gateway balloon was 80% of the diameter of the normal vessel at the distal end of the stenosis. The center has been using Gateway-Wingspan system or Apollo stents from beginning to end. We selected all cases with the same surgical methods and instruments to join the study. A prospective and observational registration study of multicenter symptomatic intracranial artery stenosis stent treatment in China showed that there was no difference in the probability of primary outcome between patients treated with balloon-mounted stent and patients treated with self-expanding stent (15).

**Table 1:** Comparation between the Gateway-Wingspan system and the Apollo stent.

	**Characteristics**			**Applicable patients**
Gateway-Wingspan system	Self-expanding stent	Bare-metal	Better flexibility in traversing curvature	Tortuous arterial access, Mori C type of lesion, a lesion with a significant mismatch in the diameter between the proximal and distal segments.
Apollo stent	Balloon-mounted stent	Bare-metal	Rigid, does not require replacement, less surgical time	Smoother arterial access, Mori A type of lesion.

According to the diameter and the length of the stenosis, the size of the Wingspan stent was selected (the stent extends at least 3 mm on both sides of the lesion). If the Apollo stent system was selected, the diameter of the Apollo stent was the same as that of the adjacent normal vessels (the smaller of the two sides of the stenosis) or slightly smaller (1:1 or 0.9:1), and the length of the stent was 1–2 mm longer than that of both sides of the target lesion. The guiding catheter was used for re-angiography.

All patients underwent computed tomography (CT) scans for bleeding or early cerebral infarction within 24 h after endovascular treatment. Dual antiplatelet therapy (clopidogrel 75 mg and aspirin 100 mg orally) and statins (atorvastatin 20 mg or rosuvastatin 10 mg orally) were generally continued for 6 months post-procedure, after which the intake of one antiplatelet agent was stopped. All medical management procedures were the same as that in the SMT group.

### Follow Up

All patients were followed up by clinic visits or telephone interviews at 7 days, 1, 6 months, 1 year, and once a year after the start of medication or surgery. Neurologists perform detailed neurological assessments, including standardized assessment [National Institutes of Health Stroke Scale (NIHSS), Modified Rankin Score (mRS), etc.] and recorded any intermittent occurrence of ischemic symptoms. Ischemic stroke in the territory of the qualifying artery, hemorrhage, or death within 1 year were recorded as primary endpoints. Stroke severity and incidence of restenosis were assessed during the follow-up period. The neurological disability at baseline was defined by mRS. The deterioration of the severity of a stroke event during follow-up was defined by NIHSS. Ischemic stroke is divided into mild (non-disabling) stroke, defined as NIHSS score worsening of 3 points or less, or severe (disabling) stroke, if the baseline NIHSS score deteriorated by more than 3 points. For patients with neurological symptoms, brain imaging examinations, including CT angiography and DSA were performed. We calculated the degree of restenosis relative to the adjacent distal normal blood vessel diameter (WASID standard). Restenosis was defined as ≥ 50%. If the patient was diagnosed with restenosis, we classified the patient as symptomatic or asymptomatic. Hemorrhagic stroke was defined as an intracerebral hemorrhage, including parenchymal, subarachnoid, or intraventricular hemorrhage, which was related to seizures, symptoms, or signs, and lasted for at least 24 h. The differences between the two groups at 7 days, 1, 6 months, and 1 year were analyzed. The stroke rates and severe stroke rates in 1–12 months between the two groups were analyzed.

### Statistical Methods

Using the SPSS 25.0 version statistical package (Armonk, NY: IBM Corp.), the measurement data were expressed as mean ± standard deviation ($\bar{x}\pm$ s). *The t-*test was used to compare the two samples (medical group and PTAS group). The chi-squared test or Fisher's exact probability method was used to compare the enumeration data. Patients from PTAS group were used as a reference group in the Cox proportional hazards model to determine hazard ratios (HRs) as effect size measures, with their 95% confidence intervals (CIs). Primary event rates were compared between the two groups using the Cox proportional hazards model adjusted by sex, age, hypertension, diabetes mellitus, coronary heart disease, smoking history, qualifying ischemic events, qualifying artery, high-density lipoprotein (HDL), LDL, and mRS. *P* < 0.05 was considered as statistically significant. HRs > 1 implied greater risk of SMT therapy and HRs <1 implied greater risk of PTAS.

## Results

**Figure 1:**
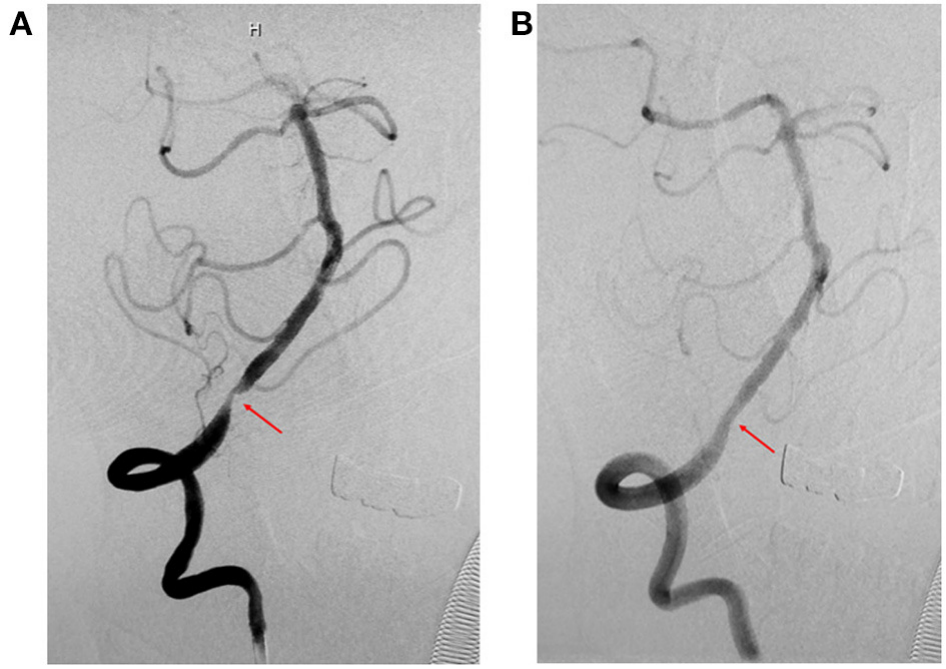
Representative images of cases of V4 before and after stenting (Apollo stent). **(A)** Digital subtraction angiography (DSA) revealed severe stenosis of the right intracranial vertebral artery (V4) before stenting (red arrow). **(B)** DSA revealed significant improvement in stenosis of the right intracranial vertebral artery (V4) after stenting (red arrow).

**Figure 2:**
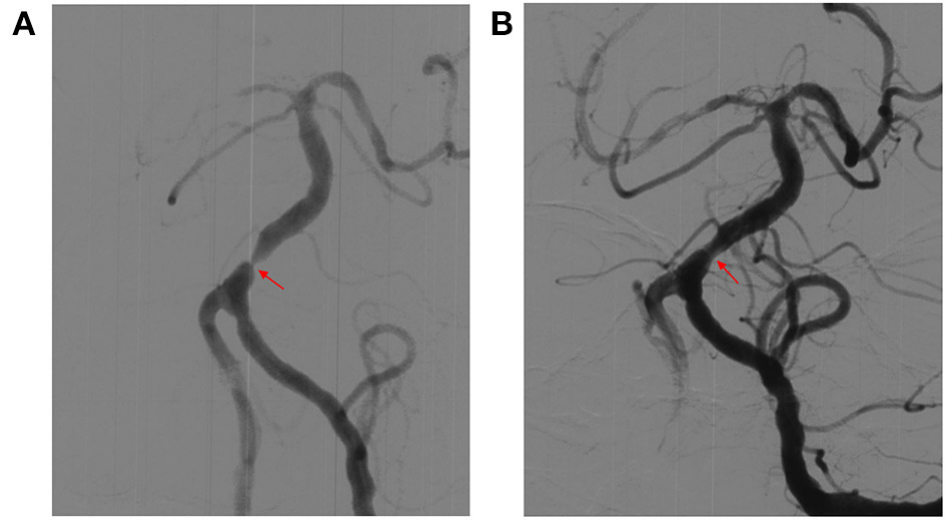
Representative images of cases of basilar artery (BA) before and after stenting (Gateway-Wingspan system). **(A)** Digital subtraction angiography (DSA) revealed severe stenosis of BA before stenting (red arrow). **(B)** DSA revealed significant improvement in stenosis of BA after stenting (red arrow).

We included 238 patients with symptomatic severe IVBS; 62 patients were treated with SMT and 176 patients underwent PTAS for IVBS. In the PTAS group, stent angioplasty was successfully performed in 174 patients (Figures 1, 2). The success rate was 98.9%. The reasons for the two patients not being able to complete surgery were tortuosity of the proximal vertebral artery, and the guide catheter could not reach the V2 segment. The mean time from the last event to stenting in the PTA cohort was 61 days.

Table 2 shows that there were no significant differences in sex, age, hypertension, diabetes mellitus, coronary heart disease, smoking history, cholesterol (HDL and LDL), or qualifying ischemic events (TIA or cerebral infarction) between the SMT and PTAS groups.

**Table 2:** Baseline characteristics of the participants between PTAS and SMT group.

**Characteristics**	**PTAS group (*n =* 174)**	**SMT group (*n =* 62)**	***p*-value**
Male, no. (%)	138 (79.3)	42 (67.7)	0.066
Age, y, mean (SD)	59.4 ± 8.7	61.0 ± 9.2	0.207
Risk factors			
Hypertension, no. (%)	150 (86.2)	55 (88.7)	0.616
Diabetes mellitus, no. (%)	62 (35.6)	27 (43.5)	0.269
Coronary heart disease, no. (%)	27 (15.5)	14 (22.6)	0.208
Smoking history			0.432
Current	56 (32.2)	15 (24.2)	
Former	28 (16.1)	13 (21)	
Never	90 (51.7)	34 (54.8)	
Cholesterol, mmol/L, mean (SD)			
High-density lipoprotein	1.0066 ± 0.216	1.0632 ± 0.243	0.088
Low-density lipoprotein	2.4474 ± 0.733	2.6195 ± 0.967	0.205
Qualifying ischemic events, no. (%)			
TIA	73 (42)	28 (45.2)	0.661
Cerebral infarction	99 (56.9)	32 (51.6)	0.472
Qualifying artery, no. (%)			0.998
BA	73 (42)	26 (41.9)	
Intracranial vertebral	101 (58)	36 (58.1)	
mRS, no. (%)			0.23
<3	165 (94.8)	56 (90.3)	
≥ 3	9 (5.2)	6 (9.7)	

*PTAS, percutaneous transluminal angioplasty and stenting; SMT, standardized medical treatment; BA, basilar artery; TIA, transient ischemic attack; mRS, modified Rankin Scale*.

**Figure 3:**
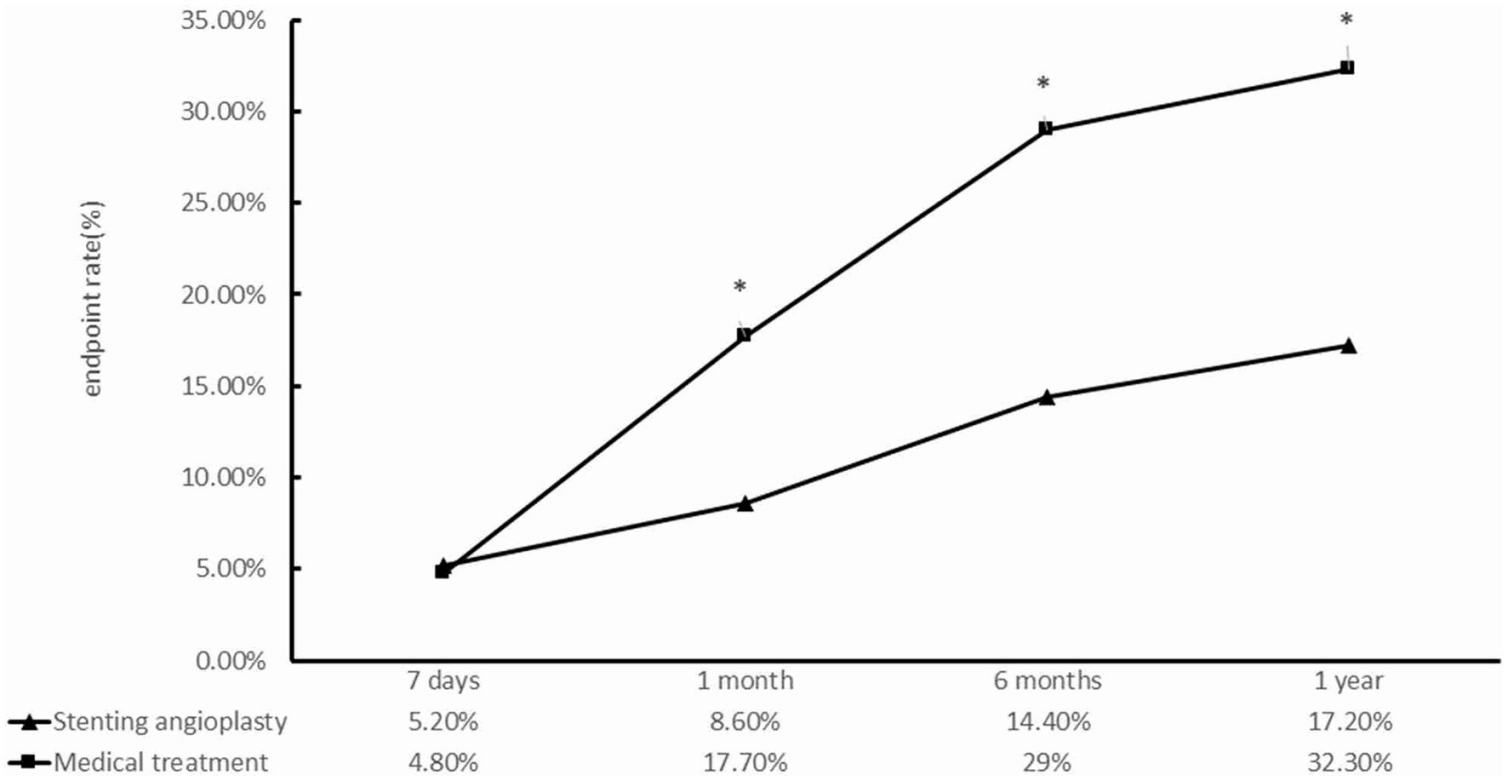
Comparison between the standardized medical treatment group and the percutaneous transluminal angioplasty and stenting group for the primary endpoints at 7 days, 1, 6 months, and 1 year. *p < 0.05.

Except for the 7th day, the SMT group had a higher frequency of primary endpoint events than did the PTAS group (Table 3, Figure 3). Figure 3 shows that the primary endpoint rates in the SMT and PTAS groups were 4.8% (3/62) and 5.2% (9/174) at the 7th day (*p* = 1.000), 17.7% (11/62) and 8.6% (15/174) at the 1st month (*p* = 0.049), 29% (18/62) and 14.4% (25/174) at the 6th month (*p* = 0.01), and 32.2% (20/62) and 17.2% (30/174) at 1st year (*p* = 0.013), respectively. Although there was no significant difference in stroke rate from the 1st month to the 1st year between the SMT group (9/62, 14.5%) and the PTAS group (13/174, 7.5%), there was a significant difference in severe stroke rates between the SMT (6/62, 9.7%) and PTAS groups (0/174) (Table 4).

**Table 3:** Primary endpoints at 7 days, 1, 6 months, and 1 year.

	**7 days**			**1 month**			**6 months**			**1 year**		
**Complications**	**PTAS group** ** (*n =* 174)**	**SMT group** ** (*n =* 62)**	***p*-Value**	**PTAS group** ** (*n =* 174)**	**SMT group** ** (*n =* 62)**	***p*-Value**	**PTAS group** ** (*n =* 174)**	**SMT group** ** (*n =* 62)**	***p*-Value**	**PTAS group** ** (*n =* 174)**	**SMT group** ** (*n =* 62)**	***p*-Value**
Stroke, no. (%)	8 (4.6)	3 (4.8)		14 (8)	11 (17.7)		23 (13.2)	18 (29)		27 (15.5)	20 (32.3)	
Ischemic stroke	5 (2.9)	2 (3.2)		11 (6.3)	10 (16.1)		20 (11.5)	17 (27.4)		24 (13.8)	19 (30.6)	
TIA	0	2 (3.2)		1 (0.6)	5 (8.1)		7 (4)	7 (11.3)		10 (5.8)	7 (11.3)	
Cerebral infarction	5 (2.9)	0		10 (5.7)	5 (8.1)		13 (7.5)	10 (16.1)		14 (8)	12 (19.3)	
Cerebral hemorrhage	3 (1.7)	1 (1.6)		3 (1.7)	1 (1.6)		3 (1.7)	1 (1.6)		3 (1.7)	1 (1.6)	
Death, no. (%)	1 (0.6)	1 (1.6)		2 (1.1)	3 (4.8)		3 (1.7)	4 (6.5)		4 (2.3)	4 (6.5)	
Stroke-related death	0	1 (1.6)		1 (0.6)	3 (4.8)		1 (0.6)	4 (6.5)		1 (0.6)	4 (6.5)	
Heart-related death	1 (0.6)	0		1 (0.6)	0		2 (1.1)	0		3 (1.7)	0	
Primary endpoints, no. (%)	9 (5.2)	3 (4.8)	1.000	15 (8.6)	11 (17.7)	0.049	25 (14.4)	18 (29)	0.01	30 (17.2)	20 (32.3)	0.013
Restenosis				3/145 (2.1%)			12/145 (8.3%)			20/145 (13.8%)		
Symptomatic restenosis				2/145 (1.4%)			7/145 (4.8%)			12/145 (8.3%)		
Asymptomatic restenosis				1/145 (0.7%)			5/145 (3.5%)			8/145 (5.5%)		

*PTAS, percutaneous transluminal angioplasty and stenting; SMT, standardized medical treatment; TIA, transient ischemic attack*.

**Table 4:** Comparison between early and delayed results in PTAS and SMT groups.

	**1-month event rate**	**1–12-month stroke rate**	1–12-month severe stroke^*^
PTAS group (*n =* 174)	14 (8%)	13 (7.5%)	0
SMT group (*n =* 62)	11 (17.7%)	9 (14.5%)	6 (9.7%)
*p*-Value	0.049	0.10	0

* *classified a stroke as a severe stroke if the baseline NIHSS score deteriorated by more than 3 points*.

*PTAS, percutaneous transluminal angioplasty and stenting; SMT, standardized medical treatment; NIHSS, National Institutes of Health Stroke Scale*.

Table 3 shows the primary end points and other major adverse events during the follow-up period in each group. On the 7th day, there was one cardiogenic death, five ischemic strokes, and three cerebral hemorrhages in the PTAS group. All ischemic strokes were caused by the responsible artery. All cerebral hemorrhages were post-operative and symptomatic. There was one fatal hemorrhage stroke and two ischemic strokes due to the responsible artery in the SMT group. From the 7th day to the 1st month, there were six patients with ischemic strokes (one fatal stroke) due to the responsible artery in the PTAS group. In the SMT group, there were eight patients with ischemic strokes (two fatal strokes) due to the responsible artery. Between 1st and 6th month, one patient died of heart disease and nine patients suffered ischemic strokes caused by the responsible artery in the PTAS group. In the SMT group, ischemic stroke in seven patients was caused by the responsible artery, including one fatal stroke. Between 6th month and 1st year, one patient died of heart disease and four patients had ischemic strokes caused by the responsible artery in the PTAS group. In the medical treatment group, two patients had ischemic strokes caused by a responsible artery, and both survived.

The mortality rates at 7th day, 1st, 6th month, and 1st year were as follows (PTAS group vs. SMT group): 0.6 vs. 1.6%, 1.1 vs. 4.8%, 1.7 vs. 6.5%, and 2.3 vs. 6.5%. Among the four deaths in the PTAS group, one resulted from the responsible artery, and the other three were cardiogenic deaths. In the SMT group, four were stroke-related deaths.

Of the 174 patients in the PTAS group, 145 underwent neurovascular imaging in the 1st year. Of 145 patients, 20 patients (13.8%) showed restenosis of the surgical segment of 50% or more. Among these patients, 12 (60%) were symptomatic and eight (40%) were asymptomatic. In symptomatic patients, ten patients resumed dual antiplatelet therapy for 3 months, and two patients underwent angioplasty alone. In asymptomatic patients, all eight patients resumed dual antiplatelet therapy for 3 months. During the 1-year follow-up period, no further stroke or death occurred in the patients who underwent angioplasty or medical treatment again (Table 3).

Table 5 shows primary event rates which were compared between the PTAS group and SMT group using the Cox proportional hazards model adjusted for sex, age, hypertension, diabetes mellitus, coronary heart disease, smoke history, qualifying ischemic events, qualifying artery, HDL, LDL, and mRS. When no adjustment factors were added, PTAS group had a lower risk of primary endpoint compared with that of SMT group (HR, 2.04; 95% CI, 1.16, 3.59; *p* = 0.014). After adjusting for other factors, the difference was still significant (HR, 2.22; 95% CI, 1.22, 4.05; *p* = 0.009). The adjusted factors had no effect on the incidence of primary endpoint events.

**Table 5:** Comparison of the primary endpoints using the Cox proportional hazards model adjusted by various factors.

		**HR (95% CI)**	***p*-Value**
Unadjusted^*^		2.04 (1.16, 3.59)	0.014
Adjusted by factors^#^	Male	1.94 (1.09, 3.43)	0.024
	Age	1.85 (1.04, 3.29)	0.036
	Hypertension	1.85 (1.04, 3.29)	0.036
	Diabetes mellitus	1.87 (1.05, 3.34)	0.034
	Coronary heart disease	1.85(1.03, 3.30)	0.039
	Smoke history	1.93 (1.07, 3.50)	0.029
	Qualifying ischemic events	1.87 (1.04, 3.39)	0.038
	High-density lipoprotein	1.95 (1.07, 3.53)	0.028
	Low-density lipoprotein	2.04 (1.12, 3.72)	0.019
	mRS	2.24 (1.23, 4.09)	0.008
	Qualifying artery	2.22 (1.22, 4.05)	0.009

* *regardless of other factors, patients in PTAS group were used as a reference group in the Cox proportional hazards model to determine hazard ratios (HRs) as effect size measures, with their 95% confidence intervals (CIs)*.

# *factors were sequentially added for adjusting. The comparison was adjusted by adding factors one by one; the last line was the adjusted result when all factors were added*.

*HR, hazard ratio; CI, confidence interval; mRS, Modified Rankin Score*.

## Discussion

In this study, we found significant differences in the primary endpoint event rates between PTAS and SMT in patients with symptomatic IVBS at 1, 6, and 12 months, which suggests that PTAS could be superior to SMT. On follow-up, the rate of severe stroke rate from the 1st to 12th month after stenting was very low at 0% in the PTAS group, while it was high to 9.7% in the SMT group. In addition, the restenosis rate of the surgical segment in the PTAS group was 13.8% in the first year, and among these patients, 60% were symptomatic restenosis, and 40% were asymptomatic restenosis.

PTAS showed an advantage in patients with symptomatic IVBS, which was inconsistent with the results of the SAMMPRIS and VISSIT trials. These studies showed that active medical treatment was superior to PTAS for patients with intracranial artery stenosis (9, 10). An analysis of the SAMMPRIS trial on the posterior artery subgroup showed that 2-year endpoint event rates were higher in the PTAS group than in the medical group (27% vs. 9.8%) (16). In the present study, the 1-year endpoint event rate was 32.2% for all patients in the medical group. The higher risk of end point event rate may be due to unsatisfactory implementation of SMT in this study. First, all patients were treated with aspirin and clopidogrel double antiplatelet aggregation. However, some studies have shown that gene mutations in Chinese people (more than 50%) affect the antiplatelet efficacy of clopidogrel (17). In this study, only individual patients were performed platelet function testing, those patients were excluded from this study in order to avoid affecting the results. Second, the patients came from all parts of Shandong Province. During follow-up, the medication of some patients could not be completely unified because the drug manufacturers were different, which may lead to a reduction in drug efficacy, and was quite common in real-world China. Third, most patients were not treated by local hospitals due to complex conditions. In contrast, in the SAMMPRIS trial, a randomized controlled trial, the vast majority of patients were non-Asians, and great efforts have been made to control the interference factors (18). This type of aggressive medical treatment is difficult to carry out in the real world. This result suggests that medical treatment needs to be strengthened, so that patients can benefit from medical treatment in the real world.

In this study, the stroke recurrence or death rate for all patients in the PTAS group was 8.6% at 30 days after the procedure, and the annual rate was 17.2%, which was consistent with the results of previous studies on posterior circulation. A meta-analysis indicated that PTAS was associated with an 8% incidence of stroke recurrence or death in patients with IVBS within 30 days after the procedure (19). A study showed that stenting for symptomatic IVBS, the primary outcome of 30-day stroke, TIA, or death was 7.2% (13). Another meta-analysis of stroke recurrence rates in symptomatic IVBS patients showed that the risk of annual stroke recurrence or death was 14.8% (3). The WEAVE trial showed that in 152 patients with symptomatic ICAS (stenosis from 70 to 99%), there were 4 (2.6%) patients with stroke, bleeding, or death events within the 72 h after the procedure (11). In the WOVEN Trial, the natural extension of the WEAVE Trial, there were 11 strokes or deaths (8.5%) in 129 patients at 1-year follow-up (12). These studies suggest that through best practice and careful patient selection, the incidence of perioperative complications in symptomatic ICAS patients who receive stent implantation can be maintained at a low level, and the long-term effect of stent implantation may be comparable to or better than drug treatment alone. In this study, the mean time from the last event to stenting in the PTA cohort was 61 days. This may be one of the reasons for the lower rate of complications in the PTAS group. A study showed that patients with delayed (> 14 days) ICAS stenting had a lower risk of long- term cerebral vascular events than those in whom the procedure was carried out <14 days of the qualifying event (20). Although there was no significant difference in stroke rates from the 1st to 12th month between the SMT (9/62, 14.5%) and PTAS groups (13/174, 7.5%), there was a significant difference in the rate of severe stroke between the PTAS (0%) and SMT groups (9.7%). The rate of severe stroke in the PTAS group showed a very low incidence of long time, as it was 0.8% in the WOVEN trial, and in the stenting arm of SAMMPRIS at 2.2% (12). This result suggests that symptomatic IVBS patients with stenting have a lower trend of delayed severe strokes than do patients with SMT. Patients with IVBS who receive stent therapy may have better long-term neurological function recovery and lower disability rates. Nevertheless, larger randomized trials are required to verify these findings.

All three cases of cerebral hemorrhage in the PTAS group occurred within 7 days after the surgery. In PTAS, the increased risk of rupture may be due to the following: (1) the thin wall of the intracranial arteries (21); (2) the intracranial arteries located in the cerebrospinal fluid of the subarachnoid cavity with no tissue around them; (3) the perforating arteries of the intracranial arteries and the possible damage of these arteries during stent placement (22); and (4) the experience of the operator is another important factor that affects the outcome of stent therapy. To reduce the risk of stroke recurrence or death, the technology needs to be improved based on the above characteristics in the future.

In the first year, the restenosis rate of the surgical segment in the PTAS group (13.8%) was similar to that in the WOVEN study (15.2%) (12). Among restenosis patients, 60% were symptomatic, which showed that in-stent restenosis was an important cause of recurrent stroke after PTAS in symptomatic IVBS patients. In future studies, the development of new technologies, new materials, or new drugs to reduce the rate of restenosis of stents may help reduce the incidence of stroke after stenting.

In this study, the duration of DAPT in the PTAS group was different from that in the SMT group. However, this discrepancy should not lead to misinterpretation of the results. A study showed that there was a tendency toward lower rate of any ischemic stroke in the patients who used DAPT beyond 90 days, but the difference was not statistically significant (23). Different from the high-dose applications of statins in Europe and America, the low doses of statins were used in this study. The results of the HPS2-THRIVE study showed that under the same dose of statin treatment, the incidence of liver adverse reactions in patients with cardiovascular disease in China was significantly higher than that in European patients, and the incidence of elevated liver enzymes was higher in European patients, and the risk of myopathy was 10 times higher than that in Europeans (24). The results of CHILLAS showed that high-intensity statins did not benefit Chinese patients more (25). Therefore, in China, moderate-intensity statins (atorvastatin 20 mg or rosuvastatin 10 mg) are used clinically.

This study was a retrospective real-world study for symptomatic IVBS in a Chinese population. It is a double-arm study with a medical group and a stent group, which has been less frequently used in previous studies. Nevertheless, this study has several limitations. First, this was a single-center retrospective study. The procedures of the medical treatment and PTAS were not random, which increased the possibility of bias. Second, the number of patients was small to be further divided into subgroups, which may have resulted in failure to investigate subgroups that benefit from medical treatment or PTAS. Although these limitations may compromise the conclusions, large multicenter randomized clinical trials may effectively confirm or refute these conclusions in the future.

In conclusion, in real-world China, PTAS for patients with symptomatic IVBS may be superior to SMT, have better long-term neurological function recovery, and lower disability rate. In the real world, medical management needs to be strengthened to benefit more patients. These findings need to be confirmed or refuted by randomized controlled clinical trials.

## Data Availability Statement

The original contributions presented in the study are included in the article/Supplementary Material, further inquiries can be directed to the corresponding author/s.

## Ethics Statement

The studies involving human participants were reviewed and approved by the ethical standards committee on human experimentation at Shandong Provincial Hospital, Cheeloo College of Medicine, Shandong University. Written informed consent for participation was not required for this study in accordance with the national legislation and the institutional requirements.

## Author Contributions

QS, JL, and XL conceived and designed the research. GL, PY, YZ, SL, and YXu acquired the data. PY, SL, YXu, and YXi analyzed and interpreted the data. GL drafted the manuscript. QS made critical revisions of the manuscript. All authors approved the final manuscript.

## Conflict of Interest

The authors declare that the research was conducted in the absence of any commercial or financial relationships that could be construed as a potential conflict of interest.

## References

[1] Holmstedt CA Turan TN Chimowitz MI. Atherosclerotic intracranial arterial stenosis: risk factors, diagnosis, and treatment. Lancet Neurol. (2013) 12:1106–14. 10.1016/S1474-4422(13)70195-9 24135208 PMC4005874

[2] Markus HS van der Worp HB Rothwell PM. Posterior circulation ischaemic stroke and transient ischaemic attack: diagnosis, investigation, and secondary prevention. Lancet Neurol. (2013) 12:989–98. 10.1016/S1474-4422(13)70211-4 24050733

[3] Abuzinadah AR Alanazy MH Almekhlafi MA Duan Y Zhu H Mazighi M. Stroke recurrence rates among patients with symptomatic intracranial vertebrobasilar stenoses: systematic review and meta-analysis. J Neurointerv Surg. (2016) 8:112–6. 10.1136/neurintsurg-2014-011458 25501448 PMC6310226

[4] Caplan LR Wityk RJ Glass TA Tapia J Pazdera L Chang HM. New England medical center posterior circulation registry. Ann Neurol. (2004) 56:389–98. 10.1002/ana.20204 15349866

[5] Gulli G Khan S Markus HS. Vertebrobasilar stenosis predicts high early recurrent stroke risk in posterior circulation stroke and TIA. Stroke. (2009) 40:2732–7. 10.1161/STROKEAHA.109.553859 19478210

[6] Chimowitz MI Lynn MJ Howlett-Smith H Stern BJ Hertzberg VS Frankel MR. Comparison of warfarin and aspirin for symptomatic intracranial arterial stenosis. N Engl J Med. (2005) 352:1305–16. 10.1056/NEJMoa043033 15800226

[7] Qureshi AI Suri MFK Ziai WC Yahia AM Mohammad Y Sen S. Stroke-free survival and its determinants in patients with symptomatic vertebrobasilar stenosis: a multicenter study. Neurosurgery. (2003) 52:1033–40. 10.1227/01.NEU.0000057744.96295.9F 12699544

[8] Voetsch B DeWitt LD Pessin MS Caplan LR. Basilar artery occlusive disease in the New England medical center posterior circulation registry. Arch Neurol. (2004) 61:496–504. 10.1001/archneur.61.4.496 15096396

[9] Derdeyn CP Chimowitz MI Lynn MJ Fiorella D Turan TN Janis LS. Aggressive medical treatment with or without stenting in high-risk patients with intracranial artery stenosis (SAMMPRIS): the final results of a randomised trial. Lancet. (2014) 383:333–41. 10.1016/S0140-6736(13)62038-3 24168957 PMC3971471

[10] Zaidat O Fitzsimmons BF Woodward BK Wang Z Killer-Oberpfalzer M Wakhloo A. Effect of a balloon-expandable intracranial stent vs medical therapy on risk of stroke in patients with symptomatic intracranial stenosis. JAMA. (2015) 313:1240–8. 10.1001/jama.2015.1693 25803346

[11] Alexander MJ Zauner A Chaloupka JC Baxter B Callison RC Gupta R. WEAVE trial: final results in 152 on-label patients. Stroke. (2019) 50:889–94. 10.1161/STROKEAHA.118.023996 31125298

[12] Alexander MJ Zauner A Gupta R Alshekhlee A Fraser JF Toth G. The WOVEN trial: wingspan one-year vascular events and neurologic outcomes. J Neurointerv Surg. (2021) 13:307–10. 10.1136/neurintsurg-2020-016208 32561658

[13] Liu L Zhao X Mo D Ma N Gao F Miao Z. Stenting for symptomatic intracranial vertebrobasilar artery stenosis: 30-day results in a high-volume stroke center. Clin Neurol Neurosurg. (2016) 143:132–8. 10.1016/j.clineuro.2016.02.029 26943722

[14] Jiang WJ Du B Hon SF Jin M Xu XT Ma N. Do patients with basilar or vertebral artery stenosis have a higher stroke incidence poststenting? J Neurointerv Surg. (2010) 2:50–4. 10.1136/jnis.2009.000356 21990559

[15] Ma N Zhang Y Shuai J Jiang C Zhu Q Chen K. Stenting for symptomatic intracranial arterial stenosis in China: 1-year outcome of a multicentre registry study. Stroke Vasc Neurol. (2018) 3:176–84. 10.1136/svn-2017-000137 30294474 PMC6169608

[16] Lutsep HL Lynn MJ Cotsonis GA Derdeyn CP Turan TN Fiorella D. Does the stenting versus aggressive medical therapy trial support stenting for subgroups with intracranial stenosis? Stroke. (2015) 46:3282–4. 10.1161/STROKEAHA.115.009846 26382173 PMC4624506

[17] Wang Y Zhao X Lin J Li H Johnston S C Lin Y. Association between CYP2C19 loss-of-function allele status and efficacy of clopidogrel for risk reduction among patients with minor stroke or transient ischemic attack. JAMA. (2016) 316:70–8. 10.1001/jama.2016.8662 27348249

[18] Chimowitz MI Lynn MJ Turan TN Fiorella D Lane BF Janis S. Design of the stenting and aggressive medical management for preventing recurrent stroke in intracranial stenosis trial. J Stroke Cerebrovasc Dis. (2011) 20:357–68. 10.1016/j.jstrokecerebrovasdis.2011.05.001 21729789 PMC3506385

[19] Mao Y Nan G. Center volume and the outcomes of percutaneous transluminal angioplasty and stenting in patients with symptomatic intracranial vertebrobasilar stenoses: a meta-analysis. PLoS ONE. (2018) 13:e0200188. 10.1371/journal.pone.0200188 29990366 PMC6039023

[20] Zhang Y Sun Y Li X Liu T Liu P Wang H. Early versus delayed stenting for intracranial atherosclerotic artery stenosis with ischemic stroke. J Neurointerv Surg. (2020) 12:274–8. 10.1136/neurintsurg-2019-015035 31285375

[21] Wilkinson IM. The vertebral artery extracranial and intracranial structure. Arch Neurol. (1972) 27:392–6. 10.1001/archneur.1972.00490170024004 5078895

[22] Markus HS Harshfield EL Compter A Kuker W Kappelle LJ Clifton A. Stenting for symptomatic vertebral artery stenosis: a preplanned pooled individual patient data analysis. Lancet Neurol. (2019) 18:666–73. 10.1016/S1474-4422(19)30149-8 31130429

[23] Abdul Rahman L Turan TN Cotsonis G Almallouhi E Holmstedt CA Chimowitz MI. Dual antiplatelet therapy beyond 90 days in symptomatic intracranial stenosis in the SAMMPRIS trial. J Stroke Cerebrovasc Dis. (2020) 29:105254. 10.1016/j.jstrokecerebrovasdis.2020.105254 32992190 PMC7686095

[24] Group HTC. HPS2-THRIVE randomized placebo-controlled trial in 25 673 high-risk patients of ER niacin/laropiprant: trial design, pre-specified muscle and liver outcomes, and reasons for stopping study treatment. Eur Heart J. (2013) 34:1279–91. 10.1093/eurheartj/eht055 23444397 PMC3640201

[25] Zhao SP Yu BL Peng DQ Huo Y. The effect of moderate-dose versus double-dose statins on patients with acute coronary syndrome in China: results of the CHILLAS trial. Atherosclerosis. (2014) 233:707–12. 10.1016/j.atherosclerosis.2013.12.003 24603217

